# The Bright Side of the Tiger: Autofluorescence Patterns in *Aedes albopictus* (Diptera, Culicidae) Male and Female Mosquitoes

**DOI:** 10.3390/molecules27030713

**Published:** 2022-01-21

**Authors:** Anna C. Croce, Francesca Scolari

**Affiliations:** 1 Institute of Molecular Genetics, Italian National Research Council (CNR), Via Abbiategrasso 207, 27100 Pavia, Italy; 2 Department of Biology & Biotechnology, University of Pavia, Via Ferrata 9, I-27100 Pavia, Italy

**Keywords:** resilin, chitin, melanin, antennae, maxillary palps, scales, imaging, spectrofluorometry, sexual dimorphism

## Abstract

Light-based events in insects deserve increasing attention for various reasons. Besides their roles in inter- and intra-specific visual communication, with biological, ecological and taxonomical implications, optical properties are also promising tools for the monitoring of insect pests and disease vectors. Among these is the Asian tiger mosquito, *Aedes albopictus*, a global arbovirus vector. Here we have focused on the autofluorescence characterization of *Ae. albopictus* adults using a combined imaging and spectrofluorometric approach. Imaging has evidenced that autofluorescence rises from specific body compartments, such as the head appendages, and the abdominal and leg scales. Spectrofluorometry has demonstrated that emission consists of a main band in the 410–600 nm region. The changes in the maximum peak position, between 430 nm and 500 nm, and in the spectral width, dependent on the target structure, indicate the presence, at variable degrees, of different fluorophores, likely resilin, chitin and melanins. The aim of this work has been to provide initial evidence on the so far largely unexplored autofluorescence of *Ae. albopictus*, to furnish new perspectives for the set-up of species- and sex-specific investigation of biological functions as well as of strategies for in-flight direct detection and surveillance of mosquito vectors.

## 1. Introduction

Autofluorescence (AF) emission in the near-UV-visible/near-IR spectral range can occur in all living organisms when irradiated with proper excitation light, depending on the presence of endogenous biomolecules with suitable chemical properties [1]. These include conjugated double bonds, aromatic, oxidized and crosslinked complex structures, with molecular energy conditions allowing electronic transitions matching with light absorption and emission. Early observations on autofluorescence from different biological substrates have paved the way to constant and innumerable studies entailing light based phenomena. Indeed, the direct involvement of endogenous fluorophores in the structure and metabolic functions of cells and tissues, causing them to act as intrinsic biomarkers, is at the basis of the development of diagnostic strategies in biomedicine, as well as of various industrial and analytical procedures with almost countless applications in many areas, from microorganisms to vegetables or animals [2].

Interest is increasingly addressed to light-based events in Arthropoda, and their various consequences [3]. The biological and ecological roles of light-based events have been related to inter- and intra-specific visual communication, including species recognition, mate finding, prey detection, camouflage and agonistic behavior [3,4]. On the other hand, insect optical properties have been suggested as a tool to sensitively monitor the presence of pest larvae in stored food or during processing [5], to detect infestations early [6], or to differentiate in situ mosquito species and sex to improve vector surveillance [7].

In this context, the fluorescence emission properties of members of this phylum of the animal kingdom are still a poorly investigated area, not only from the chemical point of view but also in terms of the photo-physical and biological aspects. Besides the characterization of the fluorescing pigments in Lepidoptera [8], and of the photonic effects of butterfly scales [9,10,11] or of the thorax and elytra cuticle in beetles [12], interest has been devoted to fluorescing components playing a functional role in mechanically active tissues. Several studies have focused on the bluish fluorescing resilin, a protein described as rubber-like due to its distinctive elastic mechanical properties, endowed by its chemical composition. In combination with other proteins or with the sclerotized or chitinous cuticle, fluorescing in the green and red spectral regions, respectively, resilin allows the accomplishment of a variety of mechanical and receptor functions [13,14,15]. In this regard, the particular ability of the antennae to play various sensory roles in detecting chemicals, as well as sound vibrations, can account for the frequent occurrence of species- and sex-specific structural variability entailing differences in the compositional material, responding to the adaptation to the different needs of mosquitoes [16,17]. For example, the in situ analysis of both mosquito and midge antennae AF highlighted the importance of the compositional material in modulating the mechanical response to vibrations, with perspectives for the set-up of sound or vibration biomimetic sensor detectors [16,17]. On the other hand, a sophisticated characterization of the optical properties of the entire body of some mosquitoes, in terms of scattering properties investigated using a multispectral and polarimetric setup, has provided promising perspectives for the species-specific detection of in-flight mosquitoes for entomological purposes and the direct surveillance of malaria vectors [7]. Although these reports suggest that natural fluorescence has an important role in insect studies, this phenomenon is still poorly investigated, especially in mosquitoes.

The Asian tiger mosquito, *Aedes albopictus* (Skuse, 1894) (Diptera, Culicidae), is an aggressive day-light biter competent for at least 20 arboviruses, including chikungunya, Zika and dengue viruses [18,19,20,21,22,23,24]. This species is a global invader because of its life history traits and the high, human-driven, propagule pressure [25]. In the absence of effective vaccines against the pathogens vectored by *Ae. albopictus*, the control of mosquito populations is essential to limit the burden of diseases. Expanding knowledge on the AF patterns of *Ae. albopictus* tissues is of relevance not only to acquire a deeper knowledge of these phenomena in the frame of the biology of the species, but also to devise innovative applications for in situ surveillance of mosquito vectors, as suggested for *Anopheles* species [7]. Currently, optical data on *Ae. albopictus* are practically limited to a study on the whole-body reflectance properties, which were shown to be sensitive to background colors when these mosquitoes were used as models to investigate technologies for light-based proximal remote sensing for the classification of biological and engineering objects [26].

The aim of this study was to: (i) describe, for the first time, the AF properties of *Ae. albopictus* adults; (ii) assess possible AF differences in sexually dimorphic traits. Mosquitoes are indeed ideal targets to explore sexual dimorphism since males and females display different behaviors enabled by specific body structures, also important for their role as pathogen vectors. Hence, particular attention has been devoted to the antennae and maxillary palps, as sexually dimorphic head appendages with key sensory roles. Moreover, we focused on abdominal and leg scales, which are common to both sexes and contribute to the typical striped pattern of the Asian tiger mosquito. The AF of these structures has been characterized by means of microscope AF imaging, to provide directly and in a label-free manner the topological distribution of the emission signal, and a microspectrofluorometric approach, expected to indicate the presence of differently fluorescing biomolecules. The results of this study provide interesting perspectives for the application of AF-based procedures to novel species- and/or sex-specific methods for mosquito control in the field.

## 2. Results

### 2.1. Autofluorescence in the Head Appendages of Male and Female Ae. albopictus Adults

**Figure 1:**
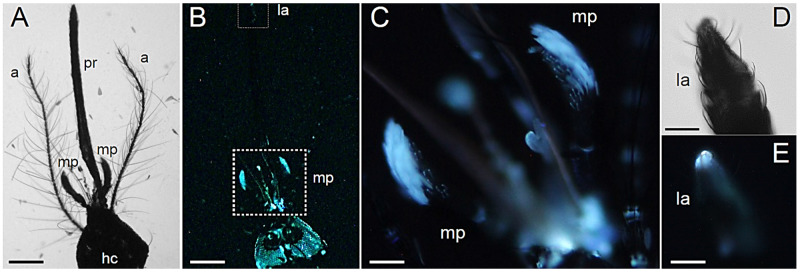
Head structures in *Ae. albopictus* adult females. (**A**) Bright field light view of the dorsal female head showing proboscis (pr), maxillary palps (mp) and antennae (a) departing from the head capsule (hc). (**B**) Fluorescent light view of the dorsal female head. Labella (la). (**C**) Fluorescent light view of the maxillary palps (mp). Bright field (**D**) and fluorescent (**E**) light view of the labella. Bars: 250 μm (**A**,**B**); 60 μm (**C**); 30 μm (**D**,**E**).

**Figure 2:**
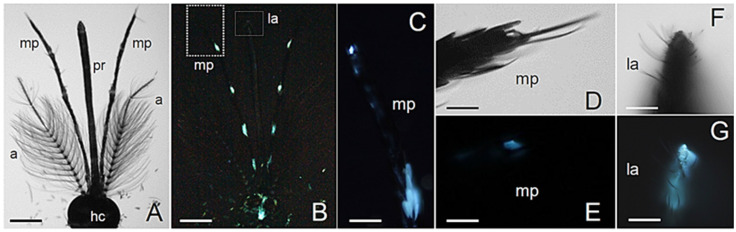
Head structures in *Ae. albopictus* adult males. (**A**) Bright field light view of the dorsal male head showing proboscis (pr), maxillary palps (mp) and antennae (a) departing from the head capsule (hc). (**B**) Fluorescent light view of the dorsal male head. Labella (la). (**C**) Fluorescent light view of the distal portion of a maxillary palp. Bright field (**D**) and fluorescent (**E**) light view of the terminus of a maxillary palp. Bright field (**F**) and fluorescent (**G**) light view of the labella. Bars: 360 μm (**A**,**B**); 90 μm (**C**); 25 μm (**D**,**E**); 40 μm (**F**,**G**).

Observations of *Ae. albopictus* male and female adult heads under bright field microscope conditions allow the appreciation of the marked morphological differences in the antennae and maxillary palps (Figure 1A and Figure 2A) related to sexual dimorphism in mating and feeding behaviors [27].

Differences emerge also in terms of AF patterns, which evidence regions with strong bluish emission (Figure 1 and Figure 2).

In both sexes, fluorescence arises from the antennae, the maxillary palps and the labella (Figure 1B and Figure 2B). In the female, the silvery-white scales present in the apex of palpomere 4 [28] display a strong bluish emission (Figure 1B,C), similarly to the very tip of the proboscis (Figure 1D,E). In the male, the maxillary palps are longer than in the female and show a strong AF in correspondence to the silvery-white scale bands located at the base of palpomeres 2 to 5 [29] (Figure 2B,C) and in the terminus of the maxillary palp (Figure 2D,E). Similar to what is observed in the female, blue emission is detectable in the very tip of the proboscis (Figure 2F,G), which is dark scaled [29].

**Figure 3:**
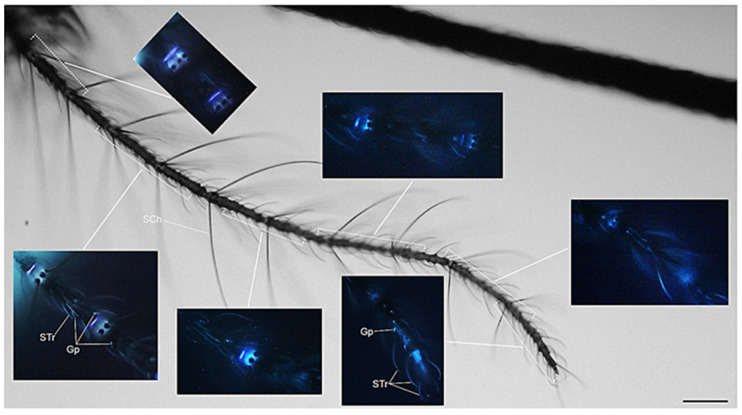
*Ae. albopictus* adult female antenna. Bright field light view of the antenna, with inserts showing the AF in the flagellomer joints and in the sensilla. Sensilla chaetica (SCh); sensilla trichoidea (STr); short grooved peg (Gp) sensilla. Bar: 100 μm.

**Figure 4:**
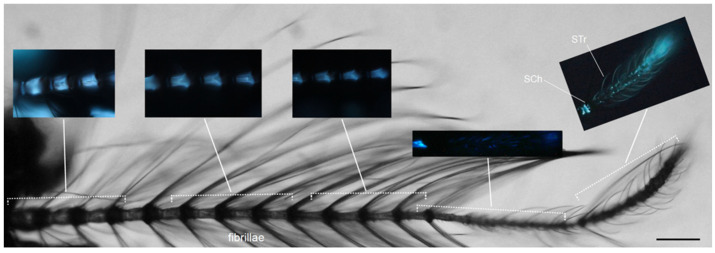
*Ae. albopictus* adult male antenna. Bright field light view of the antenna with inserts showing the AF in flagellomers 1 to 11 and in the sensilla, especially those located on the terminal flagellomer. Sensilla chaetica (SCh) arising from their sockets; sensilla trichoidea (STr). Bar: 100 μm.

When focusing on the antenna, which in both sexes consists of thirteen flagellar segments, called flagellomers [30], a sex-related pattern of fluorescence is evident both at the flagellomer joints and in the sensilla (Figure 3 and Figure 4). As to the flagellomer joints, in the female two different regions appear to be fluorescent: a thin disc and a larger cone, where sensilla chaetica arise from their sockets (Figure 3).

In the male, flagellomers 1 to 11 bear long fibrillae that are organized in a complex with the scolophores of the Johnston’s organ and are involved in hearing [30]. These eleven flagellomers are intensely fluorescent, with a blue disc distinguishable at the joints (Figure 4).

Antennal sensilla are distributed along the entire length of the female antenna and the two terminal segments in the male.

In the female, non-fluorescent sensilla chaetica insert in the flagellomer joint cones and are organized in a whorl at the base of each of flagellomers 2–13 (Figure 3). Along the flagellomers, fluorescence is visible in the sensilla trichoidea—hair-like structures distributed on each segment of the antennal flagella, where they are the primary olfactory sensilla—and in the short-grooved peg (Gp) sensilla (Figure 3).

In the male, non-fluorescent sensilla chaetica are located in whorls at the base of the terminal flagellomer, whereas the subterminal segment carries fibrillae at the base and sensilla chaetica along the shaft (Figure 4). The terminal segment shows strong fluorescence not only at the terminus, where campaniform organs are located, but also in several sensilla. In this respect, it is important to recall that short sensilla basiconica and sensilla trichodea (both long and short) are known to be distributed along the terminal flagellomer [30].

### 2.2. Autofluorescence in Ae. albopictus Body Scales

**Figure 5:**
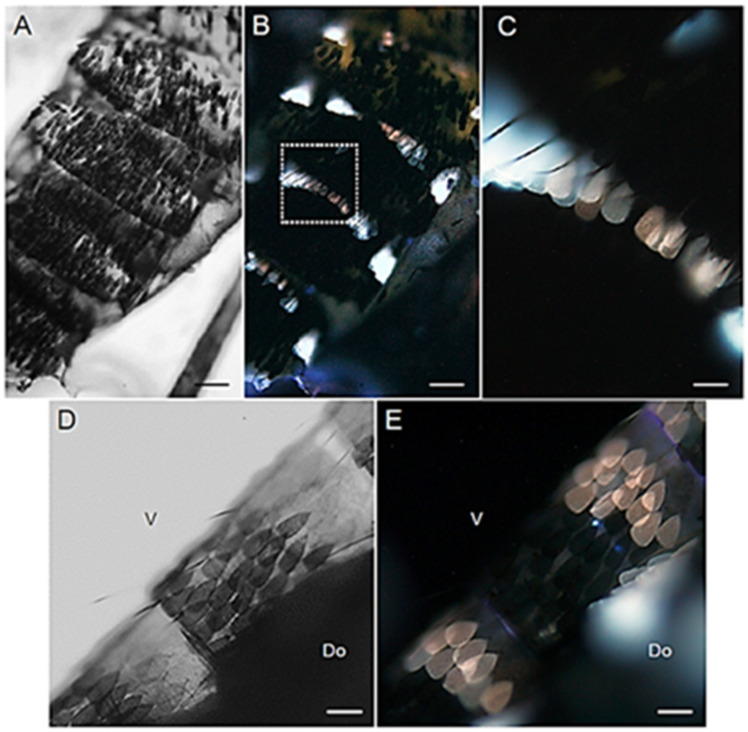
Abdominal scales in adult *Ae. albopictus* females. (**A**) Bright field and (**B**) AF view of dorsal abdominal segments. (**C**) Enlarged view of the dorsal abdominal scales indicated by the dashed box in (**B**). (**D**) Bright field and (**E**) AF view of a portion of the ventral sternites with scales. Ventral (V) and dorsal (Do) sides. Scale bars: 110 μm (**A**,**B**); 20 μm (**C**–**E**).

**Figure 6:**
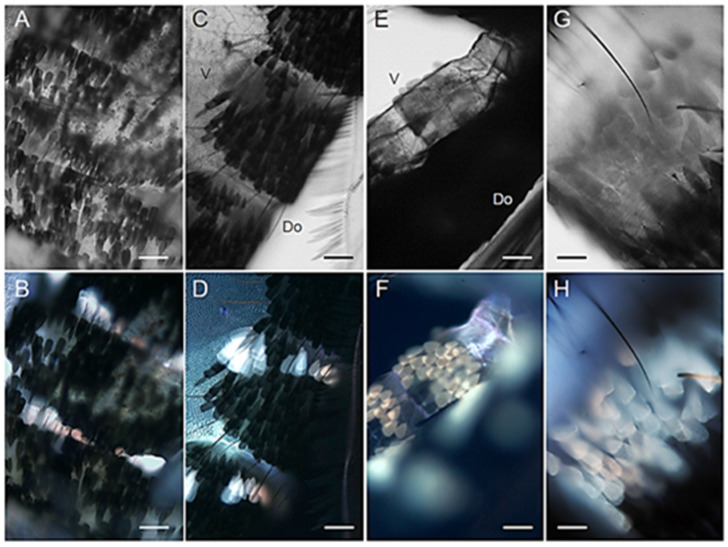
Abdominal scales in adult *Ae. albopictus* males. (**A**) Bright field and (**B**) AF view of dorsal abdominal segments. (**C**) Bright field and (**D**) AF lateral view of dorsal abdominal segments. (**E**) Bright field and (**F**) AF view of a portion of the ventral sternites with scales. (**G**) Bright field and (**H**) AF enlarged view of ventral abdominal scales. Ventral (V) and dorsal (Do) sides. Scale bars: 50 μm (**A**–**D**); 60 μm (**E**,**F**); 30 μm (**G**,**H**).

Males and females display a similar AF pattern in the organization of the scales covering the abdomen, both dorsally (tergites) and ventrally (sternites) (Figure 5 and Figure 6). Notably, the AF patches of the mosquito abdomen correspond to the white bands typical of the Asian tiger mosquito striped pattern [31]. These white scales exhibit a remarkable AF emission, either bluish or reddish, apparently depending on their localization in the tergites (Figure 5B,C and Figure 6B,D), as well as in the sternites (Figure 5E and Figure 6F,H).

**Figure 7:**
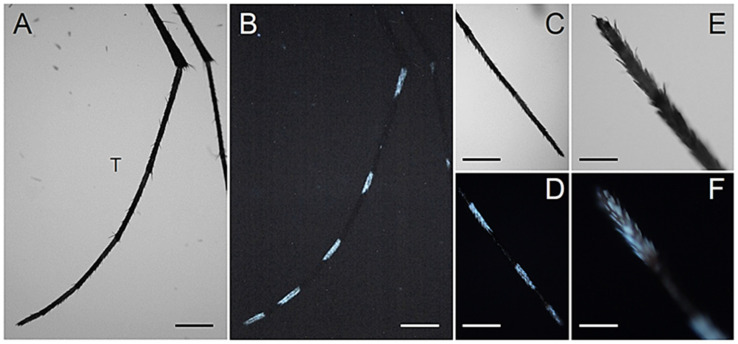
Hindleg tarsal scales in adult *Ae. albopictus* females. (**A**) Bright field and (**B**) AF view of a hindleg tarsus (T). (**C**) Bright field and (**D**) AF view of the third, fourth and fifth basal bands. (**E**) Bright field and (**F**) AF enlarged view of the fifth tarsomere. Scale bars: 400 μm (**A**,**B**); 300 μm (**C**,**D**); 80 μm (**E**,**F**).

**Figure 8:**
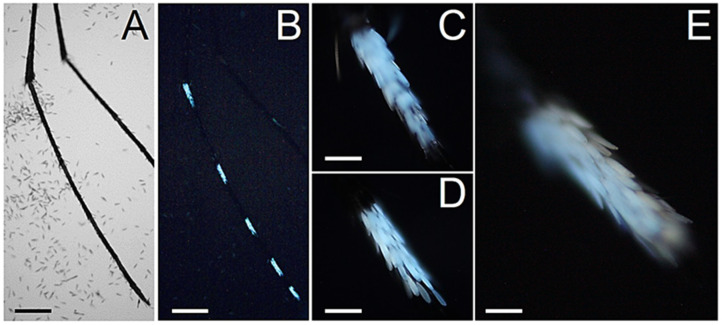
Hindleg tarsal scales in adult *Ae. albopictus* males. (**A**) Bright field and (**B**) AF view of a hindleg tarsus (T). (**C**) AF view of the fluorescent scales of tarsomere 1. (**D**) AF view of the fluorescent scales of tarsomere 3. (**E**) Enlarged view of tarsomere 5. Scale bars: 400 μm (**A**,**B**); 60 μm (**C**,**D**); 30 μm (**E**).

Considering the strong AF emission of the abdominal white scales, we also particularly focused on the observation of the hindleg tarsi, because of the abundance and size of their white bands. Indeed, the hindleg tarsi display basal white bands on tarsomeres 1-4 and an entirely white tarsomere 5 [31]. As expected, such white rings display a strong bluish AF without appreciable differences between the sexes (Figure 7 and Figure 8).

### 2.3. Spectrofluorometric Analysis

Spectrofluorometric analysis of the different fluorescing structures of *Ae. albopictus* adult bodies revealed by AF imaging indicates that emission commonly consists in a main band in the 410–600 nm spectral interval (maximum peaks ranging between 480 nm and 455 nm). Obviously, dark areas do not show appreciable signals.

**Figure 9:**
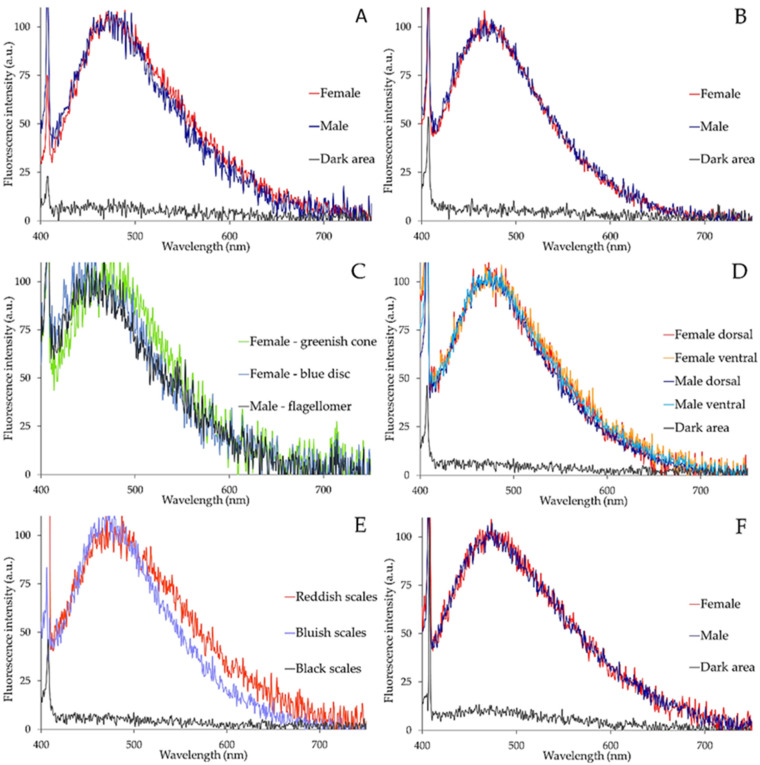
Typical examples of the AF spectra recorded from labella (**A**), maxillary palps (**B**), antennal elements (**C**), abdominal scales (**D**,**E**) and hindleg tarsi (**F**) of *Ae. albopictus* adult females and males. Spectra are normalized to the maximum peak intensity (100%) to better compare emission profiles. Spectra from females or males, or from selected structures, are identified by colors, as reported in the inlet legend on the right.

Depending on the sex and on the structure observed, spectral profiles can exhibit some differences. In the female, labella spectra show a slight widening towards longer wavelengths as compared with the male (Figure 9A), whereas the profiles of the spectra from the bluish emitting areas of the maxillary palps are very similar in the two sexes (Figure 9B). In the antennae, comparable spectral shapes are detected between the male flagellomers and the blue disc joints of the female flagellomers. The signal from the conical structures in the female flagellomer joints is instead shifted toward longer wavelengths by more than 25 nm, with a peak position at about 480 nm as compared with the peak at about 453 nm of the shorter emitting structures (Figure 9C).

Differences in spectral profiles also occur in AF emission from the scales of the abdomen.

Spectra recorded from the bluish scales of the male show a slight widening towards longer wavelengths in the 520–600 nm region in ventral as compared with dorsal scales; a similar change is observed for the spectra recorded in the female (Figure 9D). When emission spectra from bluish and reddish scales are compared, the latter show a marked widening of the spectrum towards the red region, even indicating a minor shoulder in the 520–600 nm region (Figure 9E). Finally, the fluorescing scales of the hindleg tarsi do not show remarkable differences between females and males (Figure 9F).

The parameter data characterizing the shape of the spectra shown in Figure 9 are summarized in Table 1, along with data from spectra recorded from the fore trochanter–femur joint and from the maxilla, structures commonly known to be specifically enriched in resilin and sclerotized chitin, respectively [32,33] (Figure S1).

## 3. Discussion

Autofluorescence imaging has evidenced that, in general, AF rises from distinct body compartments of the Asian tiger mosquito, *Ae. albopictus*. Spectral data, in turn, have indicated that emission consists in a major band in the 410–600 nm region, with some changes in maximum peak position and spectral width depending on the structure analyzed. These findings are likely indicating the presence of different fluorophores, as well as changes in the degree of their inter-molecular organization and/or in their possible combinations within mosquito tissues. In this regard, it is commonly known that the AF properties of various structures in arthropods are mostly attributable to proteinaceous components, namely resilin, chitin and melanin. These biomolecules are thus the most likely responsible for the AF signals detected in the body of *Ae. albopictus*.

In the mosquito head, the bluish AF observed at the antennal nodes in males and females likely reflects an enriched presence of resilin. This suggestion is corroborated by the narrow shape and short wavelength localization of AF spectra, comparable to those recorded from the foreleg trochanter–femur joint, whose richness in resilin is in turn consistent with the recognized common presence of this protein in insect leg joints [34,35]. The presence of resilin in *Ae. albopictus* antennae is also in agreement with previous reports on the antennae of the midge *Chironomus riparius*, or of the mosquitoes *Toxorhynchites brevipalpis* and *Anopheles arabiensis*, and with their functions in sound and vibration detection, with differences in sex- and species-specific morphological and mechanical properties [16,17].

Indeed, the super-elastic properties of resilin are well known to play essential mechanical roles in arthropods, assisting movement and protecting compartments engaged in movement [36,37,38].

In some mosquito species, resilin is a cuticle structural protein characterized at the genomic, transcriptomic, proteomic and ultrastructural levels [39,40]. In *Ae. aegypti*, the spermathecal duct has been shown to contain resilin, which is responsible for its capacity to stretch [41,42,43,44], and this protein has been also suggested to be present in the dorsal wall of the female labrum in different mosquitoes [45]. As to *Ae. albopictus*, to the extent of our knowledge studies confirming the specific presence of one or more resilin genes and the related functions are not available yet, apart from a preliminary BLAST search of *resilin* in the Asian tiger mosquito genome that identified potential promising candidates [46].

Our spectral data from *Ae. albopictus* antennae also evidenced a slight shift towards a longer wavelength in the signal recorded from the cones as compared with the blue discs in the female, indicating a change in resilin molecular organization and/or its mixed coexistence with other compounds, such as chitin. On the other hand, in the antennae of males, flagellomers 1 to 11 are shorter and much richer in the AF signal ascribable to resilin, likely to withstand the effort in carrying the numerous fibrillae of multiple lengths. The two terminal segments, on the contrary, appear to be more similar to the female flagellomers, and no remarkable AF signal can be appreciated from their main body, apart from numerous different sensilla along the shaft.

Actually, chitin has been already reported as a component of mosquito antennae [47], and, more generally, of insects, e.g., in coleoptera [48] and in blattodea [49].

Possible changes in the molecular features of resilin, along with the ability of this protein to coexist with chitin and additional compounds such as melanin, are acknowledged to ensure variable mechanical properties and resistance adequate to the functions of different apparatus of arthropods, as well as to the changes in fluorescing signal [13,50,51,52].

Chitin is a complex biopolymer of sugar products, acting as the structural constituent of extracellular matrices in arthropods, and which is increasingly considered for industrial and pharmaceutical sustainable applications [53,54]. A chemically-purified chitin was shown to fluoresce in the 400–600 nm spectral interval, with the maximum spectral peak at about 440 nm [55]. On the other hand, in situ image analysis applied to different arthropod species by using couples of excitation and emission filters at increasing wavelengths allowed the identification of areas with prevailing blue, green or red emission, ascribed, respectively, to resilin, to poorly sclerotized chitinous structures or to strongly sclerotized chitinous structures [13,56]. The selective detection of prevailing blue or green AF has been applied for investigations on the functional and evolutionary adaptation of arthropod structures. Examples of such studies include the analysis of the gradient in the mixing between the bluish resilin and greenish chitin accounting for the optimization of adhesive and mechanical properties of the tarsal setae of the second adhesive pad of female forelegs in the ladybird beetle *Coccinella septempunctata* [57], as well as the identification and functional characterization of antennal gustatory sensilla in the poplar leaf beetle *Crysomela populi* [15]. In this regard, the above described AF properties suggest a mixed existence of resilin and chitin in the repeated units in *Ae. albopictus* female antennal flagellum, and a richer presence of resilin in male antennae. These features are relatable to differences in mechanical properties, such as stiffness, relevant to different sensory capabilities. This suggestion is supported by a report on the sex-specific differences in relative antennal bending stiffness and vibration velocities in the two sexes of *Ae. aegypti* [58] and on the ability of mosquito females to emit sound signals to attract males and participate in bidirectional acoustic interactions [59,60].

In the maxillary palps, our AF spectra peak at slightly longer wavelengths than those from the antennal blue discs in females and flagellomers in males. In *Ae. aegypti* females, the maxillary palps have been reported to be involved in olfaction as well as mechano- and thermo-sensation. Female host-seeking is indeed known to be activated by the detection of CO_2_ by the maxillary palps [61], which vibrate during probing and in the first few seconds of blood feeding [62,63]. Interestingly, in *Ae. aegypti* CO_2_ sensitivity is similar in males and females [64], possibly to favor male nectar seeking [65]. In anopheline, based on the species- and sex-specific transcriptional profiles observed for chemosensory genes, male maxillary palps have been suggested to play a role in mate recognition [66]. On the other hand, up to now the microtrichia and scales covering both male and female maxillary palps in *Ae. aegypti* have been reported as cuticular structures without sensory functions [63]. Such a lack of sex-specific sensory functions may account for the absence of appreciable differences in AF properties, and thus in biochemical composition, between male and female palp scales in *Ae. albopictus*. In this context, and considering the morphological and functional dimorphisms between males and females, a role for the *Ae. albopictus* maxillary palp scales in improving the efficacy in chemical signal perception, already suggested for moths [67], remains an open and interesting question.

The red shift and spectral widening observed for the AF from the maxillary palps as compared with antennal nodes are even more evident in the proboscis labella, two hairy muscular lobes located at the tip of the gutter-like labium [68]. In addition to the labium, the proboscis of female mosquitoes comprises a feeding fascicle of six stylets. These structures, namely a larger and central labrum, the flattened hypopharynx with its salivary canal, a pair of long thin mandibles and a pair of needle-like maxillae, belong to the exoskeleton and consist of chitin and variable protein matrices [32]. The red shift characterizing our AF spectra may thus reflect a contribution from the sclerotized chitinous structures of *Ae. albopictus* proboscis stylets.

Additionally, the spectra recorded from the body scales show a red shift with respect to the bluish structures of the antennae. The change is less evident for the scales of the hindleg tarsi than for the abdomen. This finding can account for a different combination of resilin with other proteinaceous materials, including chitin, whose AF has been previously identified in the scales of moth and butterfly species [69].

Melanins are also likely to contribute to different extents to the AF of *Ae. albopictus* body scales. The presence of intermediate products of the melanin synthesis pathway has been already confirmed to contribute to the color of wing scales in butterflies [70,71], in addition to a recognized participation in the modulation of a wide range of physiological processes that are conserved across insects, including immunity, wound healing and protection from parasitoids and UV light [72,73]. Melanins are reddish or brown-black heterogeneous biopolymers, respectively termed pheomelanin or eumelanin, derived from the common precursor tyrosine [74]. Both eumelanin and pheomelanin strongly absorb visible light, with a continuous absorption curve decreasing exponentially from the shorter to the longer wavelengths in the visible spectral interval [75,76,77] and can exhibit distinct AF properties. In particular, synthetic eumelanin in solution at different concentrations has shown an excitation band around 360 nm when observed at emission wavelengths ranging from 450 to 570 nm [76,77], whereas a major pheomelanin component, the 6-(2-amino-2-carboxyethyl)-4-hydroxy-1,3-benzothiazole derivative, has shown a fluorescence emission spectrum in the 400–600 nm range [78]. This evidence is thus consistent with the contribution of melanins to the reddish components of the AF from the scales of *Ae. albopictus*. Finally, a possible additional contribution to the AF detected from our mosquito samples might rise from actin, a component that has been demonstrated to be essential for the development of scales, from the cylindrical shape in the pupal stages to the flattened form in the adult in *Ae. aegypti* [79].

Changes in the AF pattern of *Ae. albopictus* body scales may be thus ascribed to the presence of different biomolecules, indicating that their synthetic pathways have been adapted to meet specific needs of different insect species. Indeed, scales are known to serve a variety of functions, from species recognition and thermal regulation, to aposematism [80], and phenotypic changes in *Ae. aegypti*, including scale patterns, have been associated with seasonal variations and larval breeding sites [18,81]. However, the function of scales is still largely unknown in Diptera, including mosquitoes [80]. Despite such a knowledge gap regarding scale structure and function, a valuable practical consequence of the observed variability is that scale features can be exploited for taxonomic studies. The scale color in the Culicidae family has indeed been considered as a key characteristic to distinguish different species [28,82,83,84], and in *Ae. aegypti* the variability in abdominal scale color, ranging from white to gold, allows the differentiation of rare morphological mutants and avoids their misinterpretation as new forms or species [85].

## 4. Materials and Methods 

### 4.1. Insect Samples

*Aedes albopictus* eggs were collected using ovitraps in Northern Italy (Seniga, BS, 45°14′35.33″ N, 10°10′50.98″ E) in August and September 2021. Hatched larvae were reared on fish food pellets (Goldfish Granules, Tetra GmbH, Melle, Germany). Emerging adults were reared in a climatic chamber at 25 °C, with 60–75% relative humidity and a 12:12 h (L:D) photoperiod and maintained on a 20% sugar solution. The identity of the individuals was confirmed using the morphological keys of Rueda [28].

### 4.2. Bright Field and Fluorescence Microscopy

Individual mosquitoes were cold anesthetized and transferred to a cover slip to allow microscopic observation. The heads were dissected from cold anesthetized adults and submitted to a similar mounting procedure. Autofluorescence images were acquired by means of an Olympus BX53 fluorescence microscope (Olympus Optical Co. GmbH, Hamburg, Germany), equipped with an X-Cite 120 Q illumination system (120 W Hg vapor short arc lamp) as the fluorescence excitation source, and the UFUW optical cube for light selection (340–390 nm excitation filter, 410 nm dichromatic mirror, 420 nm long pass filter). Images were recorded using an EOS 1300D Olympus camera, by means of the Olympus objectives Plan 4× (0.10), UPlanFL 10× (0.30), UPlanFL 20× (0.50), and UPlanFL 40× (0.75). Pictures were optimized with the IrfanView 4.54 program (Irfan Skiljan copyright 1996–2020), and figure plates realized with Adobe Illustrator CC 2017 v. 21.0.0.

### 4.3. Spectrofluorimetric Analysis

Autofluorescence emission spectra were recorded from the samples under epi-illumination by means of a microspectrograph (Leitz, Wetzlar, Germany), equipped with a 100W/Hg excitation lamp (Osram, Berlin, Germany), combined with KG1-BG38 anti-thermal filters. A 366 nm band-pass interference excitation filter (FWHM 10 nm) was used to select the excitation light; fluorescence signals were recorded through a 50/50 dichroic mirror and a 390 nm long pass filter, using a 40× objective. Fluorescence emission signals were guided to a Hamamatzu PMA-12 photonic multichannel analyzer (Hamamatsu Photonics Italia Srl, Arese, Italy) by means of a fiber optic probe optically coupled to the exit slitof the microspectrograph. Spectra were recorded in the 400–750 nm interval, stored on magnetic mass memory and processed using Microsoft Excel for normalization to the maximum peak value and presentation.

## 5. Conclusions

In this study we provide, for the first time, a description of the AF patterns in *Ae. albopictus* head appendages and body scales. Our spectral data identified a number of fluorophore candidates that will be further explored through adoption of an interdisciplinary approach involving biological, physical and chemical methods. Further investigation on mosquito AF, and the chemical composition of its underlying biomolecules, is expected to improve our knowledge of insect metabolism, and of its implications in ecology and behavior. In this regard, AF patterns have demonstrated the potential to provide additional information on sexually dimorphic body compartments in *Ae. albopictus*, particularly the antennae, and can help to achieve further understanding of mating behavior and host-seeking.

Expanding knowledge on mosquito optical properties can also provide new tools for their tracing, for example contributing to develop in-flight detection and surveillance of vectors [7]. Moreover, these results pave the way to further investigation of natural fluorescence as an inter- and intra-specific communication signal and will clarify whether AF in *Ae. albopictus* may be an adaptive trait, for example, in helping to reduce detection by predators through camouflage.

## Figures and Tables

**Table 1:** Peak position and Full Width at Half Intensity Maximum (FWHM) of spectra from *Ae. albopictus* fluorescing structures.

Structure	Peak Maximum *	FWHM *
Labella	470–480 nm	140–145 nm
Maxillary palps	460–480 nm	120–130 nm
Antennal elements		
Male flagellomer/female blue disc Female greenish cone	440–470 nm 460–490 nm	123–130 nm 132–140 nm
Abdominal scales		
Bluish Reddish	460–490 nm 470–500 nm	137–145 nm 147–160 nm
Hindleg tarsi	470–480 nm	153–157 nm
Fore trochanter–femur joint	430–455 nm	134 nm
Maxilla	465–490 nm	155 nm

* Peak emission and FWHM may vary depending on the combination between different biomolecules and fluorophores.

## Data Availability

Not applicable.

## Supplementary Materials

The following is available online: Figure S1: Showing microscope images and emission spectra from a fore trochanter–femur joint and a portion of a maxilla, as typical examples of structures enriched with bluish fluorescing resilin and reddish fluorescing sclerotized chitin, respectively.

## Sample Availability

Mosquito samples are not available from the authors, because procured from the wild and analyzed as fresh material, to prevent time dependent alterations.
