# Supplementary material for: The Bright Side of the Tiger: Autofluorescence Patterns in Aedes albopictus (Diptera, Culicidae) Male and Female Mosquitoes
Source: Molecules. 2022 Jan 21;27(3):713. doi: 10.3390/molecules27030713 (PMC8839535; doi:10.3390/molecules27030713)
Supplement: Supplementary file 1 [file molecules-27-00713-s001.zip › Supplementary material_correct.pdf]

Supplementary material

The bright side of the tiger: autofluorescence patterns in *Aedes albopictus* (Diptera, Culicidae) male and female mosquitoes

Anna C. Croce*, Francesca Scolari*

1 Institute of Molecular Genetics, Italian National Research Council (CNR), Via Abbiategrasso 207, 27100 Pavia, Italy

2 Department of Biology & Biotechnology, University of Pavia, Via Ferrata 9, 27100 Pavia, Italy;

* Correspondence: croce@igm.cnr.it; Tel.: +390382986428; francesca.scolari@igm.cnr.it; Tel.: +390382986421

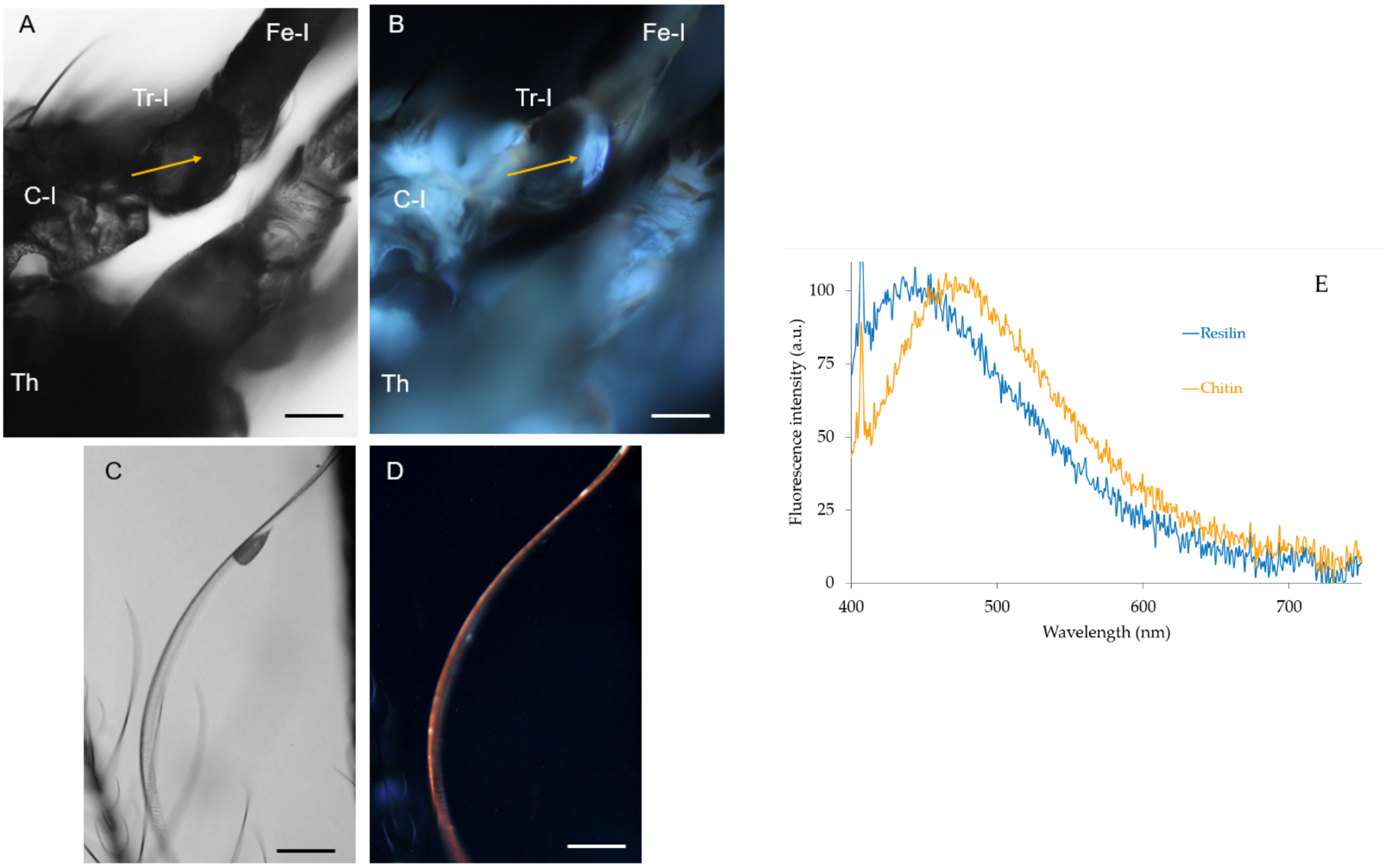

Figure S1: (**A**) Bright field and (**B**) AF view of the foretrochanter in an adult *Ae. albopictus* female. Th, thorax; C-I, forecoxa; Tr-I, foretrochanter; Fe-I, forefemur. (**C**) Bright field and (**D**) AF view of a portion of a dissected female maxilla. Bars: 100 µm (**A**, **B**); 65 µm (**C**, **D**). (**E**) Examples of AF spectra recorded from the foretrochanter-femur joint (blue), and a portion of a maxilla (orange), with prevailing emission from resilin and chitin, respectively. Spectra are normalized to the maximum peak intensity (100%).
